# Source localization of neural substrates required for generating a gamma frequency response to 40Hz GENUS light and sound stimulation

**DOI:** 10.1002/alz70859_107067

**Published:** 2025-12-26

**Authors:** Diane Chan, Monica Zheng, Ute Geigenmuller, Dimitrios Pantazis, Andrew Becker, MJ Quay, Erika Ruiz, Remi Philips, Xinran Zhang, Natalie Hahn, Samantha Yee, Brennan L Jackson, Edward S Boyden, Emery N Brown, Li‐Huei Tsai, Guillaume Leclerc

**Affiliations:** ^1^ Massachusetts Institute of Technology, Cambridge, MA USA; ^2^ Massachusetts General Hospital, Boston, MA USA; ^3^ University of Oxford, Oxford, Oxfordshire United Kingdom; ^4^ Princeton University, Princeton, NJ USA; ^5^ University of Massachusetts Amherst, Amherst, MA USA; ^6^ Howard Hughes Medical Institute, Chevy Chase, MD USA; ^7^ Broad Institute of MIT and Harvard, Cambridge, MA USA; ^8^ Picower Institute for Learning and Memory, Cambridge, MA USA

## Abstract

**Background:**

Non‐invasive gamma‐frequency light and sound stimulation at 40Hz has shown promise as a possible disease modifying therapeutic for the treatment of Alzheimer’s disease (AD). However, human neural substrates generating a gamma frequency response to Gamma Entrainment Using Sensory (GENUS) stimulation have never been shown. Here, we aim to show brain regions that generate gamma frequency oscillations in response to GENUS stimulation.

**Methods:**

We conducted an observational trial in cognitively typical subjects (n=16) undergoing magnetoencephalography (MEG) while receiving 40Hz light and sound stimulation with the GENUS device. Neurophysiological data recorded with MEG was mapped on individual T1 structural magnetic resonance (MR) images to deduce source localization of 40Hz oscillation potentiation in the brain.

**Results:**

Source localization in response to GENUS light and sound stimulation shows induced 40Hz oscillations in the appropriate sensory cortices as well as in brain regions important for associative, executive and higher order memory functions, memory consolidation, spatial orientation, and integration of sensory processes. Induced 40Hz oscillations are maximal with synchronized 40Hz light and sound stimulation as compared to light or sound stimulation alone. Interestingly, when 40Hz light and sound are anticorrelated, 40Hz oscillations are significantly reduced in the auditory and visual cortices as compared to when 40Hz light and sound are synchronized.

**Conclusions:**

For the first time, we provide evidence of target engagement and sources of gamma wave generation in the brain that are important for sensory processing, associative and executive memory functions and areas affected by AD pathology when humans are undergoing 40Hz GENUS light and sound stimulation. This data set can serve as a resource for exploring responses of neural substrates of 40Hz visual and auditory stimulation.

